# A Three-Gene Prognostic Signature Driven by an ER Stress-Associated ceRNA Network: Integrating Single-Cell Transcriptomics and Cross-Platform Validation in Hepatocellular Carcinoma

**DOI:** 10.3390/cimb48070743

**Published:** 2026-07-21

**Authors:** Qingping Shi, Shuang Gao, Beiyan Chen, Mingli Shen, Jieru Han

**Affiliations:** School of Basic Medical Sciences, Heilongjiang University of Chinese Medicine, Harbin 150040, China; sqp17326705862@163.com (Q.S.); gaoshuangacc@163.com (S.G.); chenbeiyanabc@163.com (B.C.); 18309474893@163.com (M.S.)

**Keywords:** hepatocellular carcinoma, endoplasmic reticulum stress, ceRNA network, prognostic signature, tumor microenvironment, single-cell transcriptomics

## Abstract

The progression and immune escape of HCC are closely regulated by endoplasmic reticulum stress (ERS). However, the associated ceRNA regulatory networks and their prognostic value remain to be systematically elucidated. Here, we sought to establish a prognostic signature derived from an ERS-associated ceRNA network and to investigate its relationship with the tumor immune microenvironment. We integrated TCGA-LIHC transcriptomic data with the MSigDB ERS gene set to identify ERS-associated differentially expressed genes and construct a ceRNA regulatory network. Using a forward search strategy with 10-fold cross-validation, we screened candidate genes to select the optimal prognostic combination and constructed a multigene Cox regression signature. External validation was performed in the independent microarray cohort GSE14520. By integrating single-cell transcriptomics, CIBERSORT, ESTIMATE, TIDE, and drug sensitivity analyses, we revealed immune microenvironment characteristics associated with this signature. Based on the ceRNA network’s eight core ERS mRNAs, an optimal three-gene signature comprising *STC2*, *CKS1B*, and *PSAT1* was selected via forward search. The signature demonstrated robust prognostic discrimination in the TCGA training cohort (C-index = 0.653) and was independently corroborated in the external GSE14520 dataset (C-index = 0.584, log-rank *p* = 0.008). The signature was confirmed as an independent prognostic indicator by multivariable Cox regression. Functional enrichment analysis demonstrated a marked accumulation of cell-cycle-related pathways in the high-risk group, notably DNA replication and the spindle assembly checkpoint. Single-cell transcriptomic profiling showed that *STC2* and *CKS1B* were predominantly expressed by tumor epithelial cells, whereas *CCL2* and *ATF3* were mainly detected in macrophages and fibroblasts. Drug sensitivity analysis indicated that the high-risk group was more sensitive to drugs such as docetaxel and AZD5582, consistent with the upregulation of proliferation pathways in this group; in the low-risk group, VE-822 exhibited selective sensitivity. This study established a three-gene prognostic signature based on the ERS-associated ceRNA network. The signature demonstrated robust prognostic stratification capabilities in cross-platform validation and revealed molecular characteristics centered on uncontrolled cell-cycle progression, as well as an immunosuppressive microenvironment, in the high-risk group, providing an exploratory tool for prognostic assessment and treatment strategy selection in hepatocellular carcinoma (HCC).

## 1. Introduction

Liver cancer accounted for an estimated 865,000 incident cases worldwide in 2022, making it the sixth most common malignancy. With roughly 757,948 attributable deaths, it ranked as the third leading cause of cancer-related mortality, surpassed only by lung and colorectal cancers [1]. It is the second leading cause of cancer death among men, with significant gender disparities: in most regions of the world, incidence and mortality rates among men are two to three times higher than those among women [1]. Primary liver cancer primarily includes hepatocellular carcinoma (HCC) (accounting for 75–85% of cases) and intrahepatic cholangiocarcinoma (10–15%); chronic HBV or HCV infection accounts for 21% to 55% of global HCC cases [2,3]. HCC survival is highly stage-dependent: the 5-year survival rate is approximately 36% for patients diagnosed at an early stage but falls to just 13% for those with advanced metastatic disease [4]. Although surgical resection remains the most effective curative option, offering a postoperative 5-year survival of 40–50% [5], the majority of individuals already present with intermediate- or advanced-stage HCC at initial diagnosis, and merely 30–40% are candidates for surgery [6]. Radiofrequency ablation is also considered an effective treatment, but its 5-year and 10-year recurrence rates are 74.8% and 80.8%, respectively [7]. Regarding drug therapy, although various systemic chemotherapeutic agents have been approved for treatment and can prolong survival in HCC patients, their therapeutic efficacy is suboptimal because the baseline prognosis for patients in the intermediate-to-late stages is extremely poor [8]. This poor prognosis is primarily attributed to delayed diagnosis, high recurrence rates, and the lack of reliable biomarkers for early risk stratification. Therefore, there is an urgent need to establish reliable prognostic markers and elucidate their underlying molecular mechanisms; identifying and validating reliable and easily quantifiable HCC biomarkers is crucial for addressing the growing disease burden [9].

When misfolded proteins accumulate in the endoplasmic reticulum, they elicit the unfolded protein response (UPR)—a signaling pathway that, upon activation, drives a global transcriptional adaptation to align the organelle’s folding machinery with the needs of the cell [10]. The ER may experience an imbalance between protein folding demand and its folding capacity due to physiological stresses—such as increased secretory load—or pathological stresses—such as the presence of mutant proteins that cannot be properly folded within the ER—thereby triggering endoplasmic reticulum stress (ERS) [11]. Heightened requirements for protein synthesis in tumor cells provoke ERS, which in turn triggers the UPR to preserve ER homeostasis [11]. Under mild-to-moderate ERS conditions, the UPR tends to eliminate misfolded or unfolded proteins to restore ER homeostasis; this is referred to as the “adaptive” UPR. However, under prolonged ERS, the UPR, due to excessive activation, triggers intrinsic apoptotic pathways, a process known as the “dysadaptive” UPR [10]. A trio of ER-membrane-resident proteins—PERK (PKR-like endoplasmic reticulum kinase), IRE1α (inositol-requiring enzyme 1α), and ATF6 (activating transcription factor 6)—orchestrate the UPR-signaling cascade [12]. Under non-stressed conditions, they are held in a dormant state via physical binding to the chaperone GRP78 (glucose-regulated protein 78) [13]. Upon elevated ER stress, GRP78 dissociates, thereby unleashing downstream UPR signaling. The magnitude and duration of UPR activation dictate the cell’s fate, tipping the balance between pro-survival and pro-apoptotic outcomes [14]. While modest ER stress fosters malignant traits such as increased proliferation, angiogenesis, chemoresistance, and metastatic capacity, intense ER stress activates cell death programs [10]. Moreover, growing evidence implicates severe ER stress and UPR dysregulation as key contributors to the pathogenesis of liver disease [15].

In hepatocellular carcinoma, the adaptive UPR actively promotes malignant progression. As comprehensively reviewed by Goyal et al. [10,11], dysregulated ER stress responses contribute to multiple facets of HCC pathogenesis, including apoptosis resistance, enhanced proliferation, invasion, and metastasis. The interplay between ER stress and hypoxia further fuels sorafenib resistance and tumor invasion, as highlighted by Méndez-Blanco et al. [16]. Notably, the ER chaperone GRP78 is overexpressed in sorafenib-resistant HCC cells compared to sensitive cells, and its targeted inhibition can reverse drug resistance and suppress invasion [17]. Hence, the ERS/UPR pathway is not merely an adaptive reaction to proteotoxic stress but a key driver of proliferation, invasion, metastasis, and therapeutic resistance in HCC.

Under ER stress, tumor cells undergo a profound remodeling of the non-coding RNA (ncRNA) transcriptome. Regulatory ncRNAs encompass diverse species, including microRNAs (miRNAs; 20–24 nt), PIWI-interacting RNAs (piRNAs), small interfering RNAs (siRNAs), and long non-coding RNAs (lncRNAs; >200 nt) [18]. Among these, lncRNAs are a highly heterogeneous class of transcripts: most lncRNAs are transcribed by RNA polymerase II and undergo 5′ capping, 3′ polyadenylation, and splicing, thereby exhibiting “mRNA-like” characteristics, whereas some lncRNAs transcribed by RNA polymerase I/III or derived from introns and repetitive sequences lack these classical modifications [19,20]. Compared to protein-coding genes, lncRNAs can be classified into several types based on their genomic location, specifically intergenic, antisense, intronic, and pseudogene-derived types, with some pseudogenes retaining partial function [21]. LncRNAs fulfill their regulatory roles via multiple routes—acting as transcriptional and post-transcriptional modulators of gene expression, serving as architectural scaffolds within the nucleus and cytoplasm, and participating in signaling through RNA–protein, RNA–RNA, and RNA–DNA interactions [22]. It is precisely this functional diversity that enables them to promote tumor progression in various cancers by regulating the unfolded protein response (UPR) pathway [12,23].

One of the core mechanisms by which lncRNAs exert their regulatory functions is through their role as competitive endogenous RNAs (ceRNAs) [23]. The ceRNA hypothesis posits that lncRNAs can competitively bind to miRNAs—acting like “molecular sponges”—by sharing the same miRNA response elements, thereby relieving miRNA-mediated suppression of downstream target mRNAs [24]. Extensive research in hepatocellular carcinoma has demonstrated that lncRNA–miRNA–mRNA ceRNA networks are pivotal in tumorigenesis and disease progression; for instance, the lncRNA *SNHG6* acts as a molecular sponge for *miR-204-5p*, relieving its suppression of *E2F1*, which in turn drives the G1-S phase transition and contributes to HCC development [25]. HOTAIR silences miR-122 through DNMT-mediated DNA methylation, upregulates Cyclin G1 expression, and is associated with sorafenib resistance [26,27]. Furthermore, recent studies have revealed that various lncRNAs participate in HCC progression and drug resistance by directly regulating the endoplasmic reticulum stress (ERS) signaling pathway. For instance, GAS5 inhibits HepG2 cell growth by activating the CHOP-dependent ERS pathway, whereas the *PERK/ATF4/CHOP* axis drives GOLGA2P10 transcription to confer apoptotic resistance to tumor cells [28,29]. However, these studies have largely focused on unidirectional regulation of ERS signaling by lncRNAs; the value of lncRNAs acting as ceRNAs to systematically regulate ERS-related gene networks via a sponge mechanism in HCC prognosis remains far from fully elucidated. Therefore, integrating ceRNA regulatory mechanisms with ERS biology to construct an ERS-related ceRNA prognostic model is expected to provide a new perspective for elucidating HCC molecular heterogeneity and achieving precise risk stratification.

## 2. Methods

### 2.1. Data Acquisition and Preprocessing

Multi-omics expression profiles—covering mRNA, lncRNA, and miRNA—along with corresponding clinical information for hepatocellular carcinoma cases were retrieved from the TCGA data portal (https://portal.gdc.cancer.gov) [30]. The TCGA-LIHC cohort provided 374 tumor specimens and 50 matched adjacent normal tissues. Raw expression values were normalized to transcripts per million (TPM) followed by log_2_ transformation. For independent validation, the GSE14520 dataset, comprising 221 samples profiled on the Affymetrix platform [31], was retrieved from the GEO database and processed with the limma package for background correction and quantile normalization [32]. The single-cell transcriptomic dataset GSE149614 (34,782 cells retained after quality control) was processed using the Seurat (v5) workflow. After quality control (retaining cells with 200–6000 detected genes and a mitochondrial gene proportion <15%) and log-normalization, dimension reduction and clustering were performed via PCA and UMAP. Cell types were identified by SingleR using annotations from the Human Primary Cell Atlas, ultimately identifying 8 major cell types [33]. Drug sensitivity data (IC_50_ values for 286 compounds) were obtained from the GDSC database [34]. Genes associated with endoplasmic reticulum stress (ERS) were sourced from the Hallmark collection of the Molecular Signatures Database (MSigDB).

### 2.2. Differential Expression Analysis and ceRNA Network Construction

Differentially expressed genes (DEGs) between tumor and adjacent normal samples were identified using the limma-voom pipeline, applying thresholds of |log_2_FC| > 1 and FDR < 0.05 [32]. The intersection of the DEGs and the ERS gene set was calculated, yielding 8 ERS-associated differentially expressed mRNAs (*ATF3*, *STC2*, *CCL2*, *CKS1B*, *PSAT1*, *H2AX*, *IGFBP1*, *IFIT1*). We predicted lncRNA–miRNA and miRNA–mRNA interactions using the miRcode, starBase, and TargetScan databases, constructed a ceRNA network, and visualized it using Cytoscape v3.8.0 [35].

### 2.3. Construction and Validation of the Prognostic Model

Using the 8 ERS-related DEGs as candidates, an optimal gene combination was identified through a forward search strategy with 10-fold cross-validation. Using Harrell’s C-index as the selection criterion, one gene was added in each iteration to maximize the cross-validation C-index until no further improvement was possible (increment < 0.001). Ultimately, a 3-gene signature consisting of *STC2*, *CKS1B*, and *PSAT1* was selected, with a cross-validation C-index of 0.649. A multivariate Cox proportional hazards model was constructed on the TCGA training cohort to derive individual risk scores, defined as: RiskScore = β_1_ × *STC2* + β_2_ × *CKS1B* + β_3_ × *PSAT1*. The study population was divided into two groups at the median risk score, and Kaplan–Meier survival estimates were generated and statistically compared via the log-rank test to determine whether survival diverged between risk strata. The model’s discriminatory ability was quantified using Harrell’s C-index and time-dependent AUC values at 1-, 3-, and 5-year time points, as implemented in the timeROC R package [36]. In addition, we constructed scatter plots, time-dependent ROC curves, and calibration curves using the rms package.

External validation was performed on the independent microarray cohort GSE14520 (*n* = 221). Given that the *lncRNA AC145207.5* and the gene *H2AX*, both identified in the initial ceRNA network screening, were not represented on the GSE14520 platform, the final 3-gene signature (*STC2*, *CKS1B*, *PSAT1*) provided complete coverage in both cohorts, ensuring consistency in cross-platform validation.

### 2.4. Immune Infiltration Analysis

Immune infiltration profiles were characterized by applying CIBERSORT to estimate the proportions of 22 immune cell types in TCGA samples [37], while immune scores and stromal scores were derived from ssGSEA analysis using the GSVA R package [38,39]. The TIDE algorithm was used to assess tumor immune evasion potential [40]. The Wilcoxon rank-sum test was applied to evaluate differences between the risk groups, adopting *p* < 0.05 as the cutoff for statistical significance. Immune-related analyses were all conducted in the context of the high- and low-risk strata derived from the final 3-gene prognostic classifier.

### 2.5. Machine Learning Comparison and Feature Importance

Five machine learning algorithms (CoxPH, LASSO, Ridge, ElasticNet, and random survival forest) were used to compare prognostic prediction performance via 10-fold cross-validation. RandomForestSRC was used to assess variable importance (VIMP) for the final three genes, and partial dependence plots (PDPs) were generated to illustrate the marginal effects of the risk score on 1-year mortality.

### 2.6. Drug Sensitivity Analysis

Based on the GDSC database, Ridge regression was applied to predict IC_50_ values for 286 drugs in TCGA samples [34]. Group-wise comparisons of IC_50_ values were conducted via the Wilcoxon rank-sum test, and the corresponding log_2_ fold change was computed as LogFC = log_2_(low-risk mean/high-risk mean). The screening criteria were FDR < 0.05 and |LogFC| > 0.15 to identify drugs with significantly altered sensitivity in the high-risk group.

### 2.7. Single-Cell Transcriptome Analysis

Quality-controlled single-cell data were processed by selecting 2000 highly variable genes, followed by PCA. UMAP visualization and graph-based clustering (resolution = 0.8) were subsequently carried out on the basis of the leading 20 principal components [41]. Automatic cell type annotation was conducted using SingleR and referencing the Human Primary Cell Atlas, ultimately identifying eight major cell types [42].

### 2.8. MuSiC Deconvolution and Immune–Risk Association

A balanced single-cell reference set (up to 300 cells per category) was constructed, and the MuSiC algorithm was used to decompose the TCGA bulk expression matrix into the relative proportions of the eight cell types [43]. Spearman rank correlation was used to evaluate the relationship between individual cell-type fractions and the risk score. Their independence as prognostic factors was confirmed through univariate and multivariable Cox regression. The multivariable model incorporated adjustment for age, sex, tumor stage, and histological grade.

### 2.9. Gene Set Enrichment Analysis (GSEA)

A log_2_ fold-change-ranked gene list was analyzed for pathway enrichment using clusterProfiler [44] against custom GMT files (KEGG MEDICUS, GO BP, Hallmark), with significance thresholds of FDR < 0.05, |NES| > 1.5, and 1000 random permutations.

### 2.10. Statistical Analysis

Statistical computations were conducted in R (version 4.6.0). Between-group comparisons of continuous variables were assessed using Student’s *t*-test or the Wilcoxon rank-sum test, whereas the chi-square test was applied to categorical data. The Benjamini–Hochberg method was used for multiple-comparison correction. Survival analysis was performed using the survival and survminer packages, and time-dependent ROC analysis was performed using timeROC [37]. A two-tailed *p* < 0.05 was regarded as the threshold for statistical significance.

### 2.11. Single-Cell Metabolic Pathway Activity Analysis

The AUCell algorithm was used to score metabolic pathway activity in single-cell transcriptomic data [45]. Gene sets for metabolism-related pathways were obtained from the KEGG MEDICUS collection, which was retrieved from the Molecular Signatures Database (MSigDB). Based on the standardized expression matrix of Seurat objects, we constructed a gene ranking and calculated the AUC values for each cell in each metabolic pathway. The metabolic activity scores were added to the metadata (meta.data) of the Seurat objects, and dot plots were used to visualize differences in the activity of key metabolic pathways—such as glycolysis, fatty acid oxidation, and oxidative phosphorylation—among different cell types [44].

### 2.12. Construction of the Metabolic–Immune Interaction Heatmap

To assess the potential association between metabolic reprogramming and immune infiltration, a metabolic–immune interaction matrix was constructed. Standardized enrichment scores (NESs) for selected metabolic pathways were extracted from the GSEA enrichment analysis results, while the Spearman correlation coefficients between the immune cell proportions obtained via MuSiC deconvolution and the risk scores were also utilized. The NES values of metabolic pathways were multiplied by the correlation coefficients of immune cell proportions to generate an interaction score matrix. A heatmap was then plotted using the pheatmap package to visualize the synergistic or antagonistic relationships between metabolic pathway activity and immune cell infiltration in the high-risk and low-risk groups [43].

## 3. Results

### 3.1. Differential Expression Analysis and Identification of Genes Associated with Endoplasmic Reticulum Stress

**Figure 1:**
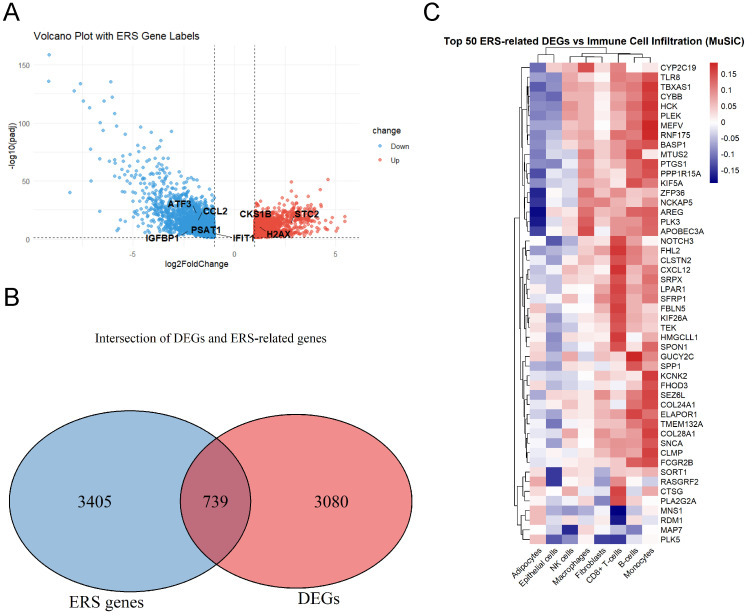
Screening of endoplasmic reticulum stress-associated differentially expressed genes in HCC and their immune relevance. (**A**) Genes differentially expressed between HCC and normal tissues are presented in a volcano plot, where up-regulated, down-regulated, and non-significant genes are colored red, blue, and gray, respectively. (**B**) Venn diagram illustrating the overlap between the full set of DEGs and endoplasmic reticulum stress (ERS)-related genes. (**C**) 50 ERS-associated DEGs exhibiting the strongest correlations and immune cell fractions estimated by MuSiC deconvolution. Red denotes positive correlations; blue denotes negative correlations.

To detect transcriptional alterations in HCC, mRNA expression profiles of 374 tumor and 50 normal tissues from TCGA-LIHC were subjected to differential analysis. In total, 3819 DEGs were detected, comprising 1504 up-regulated and 2315 down-regulated genes. A volcano plot illustrates the distribution of all DEGs and highlights eight core ERS-associated differentially expressed mRNAs selected for subsequent analysis; these genes all exhibited significant differential expression and met the criteria of |log2FC| > 1 and FDR < 0.05 (Figure 1A). By intersecting the aforementioned DEGs with the ERS-related gene set integrated from the MSigDB Hallmark collection, 739 ERS-related differentially expressed genes were identified (Figure 1B). Subsequently, Spearman correlation analysis was performed to assess the association between these ERS-related DEGs and the abundance of eight immune cell types estimated by MuSiC. A heatmap displays the 50 ERS-associated DEGs with the strongest correlations; the results show that multiple genes exhibit significant positive or negative correlations with immune cell populations such as macrophages and CD8^+^ T cells (Figure 1C), suggesting that these ERS-related genes may influence HCC progression by regulating the immune microenvironment.

### 3.2. Construction of the ceRNA Network

**Figure 2:**
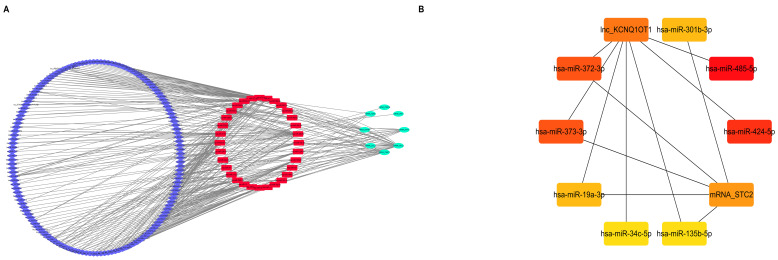
Construction of the endoplasmic reticulum stress-related ceRNA network and its core regulatory subnetwork. (**A**): The overall ceRNA network constructed based on ERS-related lncRNA–miRNA–mRNA interactions. Blue represents lncRNA; red represents miRNA; cyan represents mRNA. (**B**): The core subnetwork highlights lnc-*KCNQ1OT1* as a key “sponge” molecule that regulates multiple miRNAs targeting the ERS-related gene *STC2*.

With the aim of exploring the post-transcriptional regulation of ERS-related differentially expressed genes in HCC, a ceRNA-based lncRNA–miRNA–mRNA regulatory network was established. miRNAs that bind to ERS-associated differentially expressed mRNAs were predicted using the miRcode, starBase, and TargetScan databases, and lncRNAs containing binding sites for these miRNAs were further identified to construct the lncRNA–miRNA–mRNA regulatory network. Ultimately, the constructed ceRNA network comprised 145 lncRNA nodes, 32 miRNA nodes, eight mRNA nodes, and 360 regulatory edges (Figure 2A). Network visualization and topological analysis were performed using Cytoscape to identify core subnetworks with a high degree. The results showed that the lncRNA KCNQ1OT1 can act as a sponge to bind multiple miRNAs, including miR-424-5p and miR-485-5p, and indirectly regulate the expression of *STC2*, a key ERS gene, through these miRNAs (Figure 2B). This highly interconnected ceRNA subnetwork suggests that lncRNAs such as KCNQ1OT1 may broadly regulate the ERS pathway via a sponge mechanism, thereby playing a crucial role in HCC progression. The eight ERS-related mRNAs identified in the ceRNA network served as candidate genes for the construction of a prognostic signature using a forward search strategy.

### 3.3. Construction of a Streamlined Three-Gene Prognostic Signature via Forward Search

**Figure 3:**
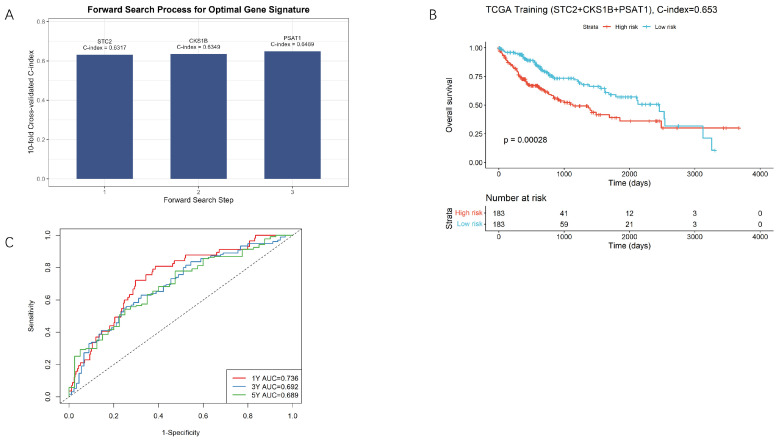
Prognostic performance of the three-gene model in the TCGA training cohort. (**A**) 10-fold cross-validation forward search showing sequential improvement in C-index upon adding *STC2* (0.6317), *CKS1B* (0.6349), and *PSAT1* (0.6489). (**B**) Survival analysis stratified by the median risk score revealed a significant separation between the high-risk (*n* = 183) and low-risk (*n* = 183) groups (log-rank *p* = 0.00028). (**C**) Prognostic accuracy over time assessed by time-dependent AUC analysis, yielding 0.736, 0.692, and 0.689 for 1-, 3-, and 5-year overall survival.

To ensure the signature’s applicability across platforms, we excluded H2AX as its corresponding probe was absent from the GSE14520 microarray platform, leaving seven genes for the forward search. Through a 10-fold cross-validation forward search with Harrell’s C-index as the selection criterion, the genes *STC2* (C-index = 0.6317), *CKS1B* (C-index = 0.6349), and *PSAT1* (C-index = 0.6489) were sequentially incorporated, ultimately identifying the optimal three-gene signature consisting of *STC2*, *CKS1B*, and *PSAT1* (cross-validation C-index = 0.6489; Figure 3A). The entire TCGA training cohort (*n* = 366) was used to fit the Cox model, achieving a Harrell’s C-index of 0.653. Using the median risk score, patients were categorized into high-risk (*n* = 183) and low-risk (*n* = 183) subsets; a significant reduction in overall survival was observed in the high-risk group (log-rank *p* = 0.00028; Figure 3B). The time-dependent AUC reached 0.736 at 1 year, 0.692 at 3 years, and 0.689 at 5 years (Figure 3C).

### 3.4. Independent Prognostic Value of the Three-Gene Signature

**Figure 4:**
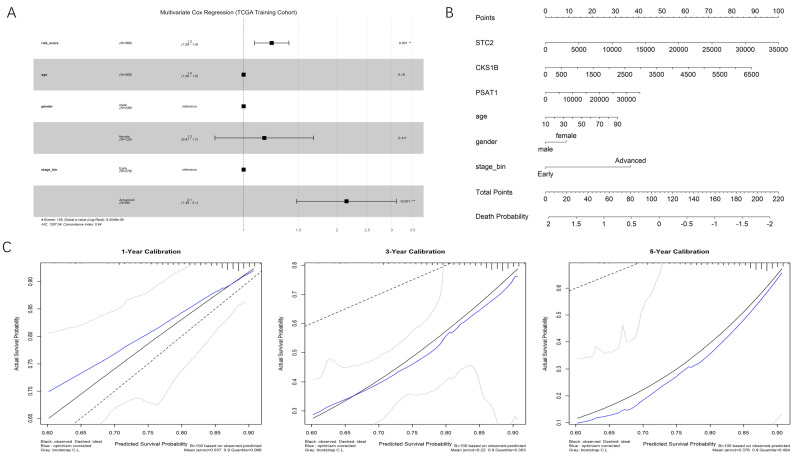
Independent prognostic assessment of the 3-gene signature. (**A**) Forest plot derived from multivariate Cox analysis, which demonstrated that the risk score retained independent prognostic significance independent of age, sex, and tumor stage. Asterisks denote statistical significance. (**B**) A nomogram combining the 3-gene classifier and clinical covariates. Tumor stage carried the greatest weight among all variables, as reflected by its longest segment. (**C**) Nomogram calibration curve showing high consistency between predicted and actual survival probabilities.

**Figure 5:**
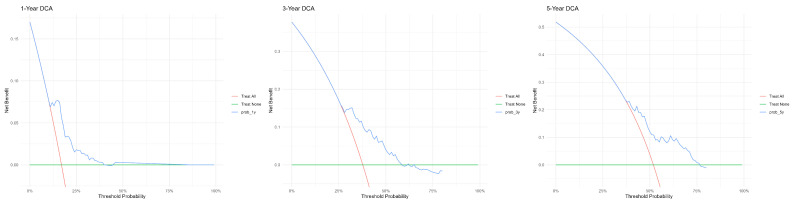
Clinical net benefit assessment by decision curve analysis. Decision curves evaluating the 3-gene signature for 1-, 3-, and 5-year overall survival prediction. At 5 years, the model provided the highest net benefit, outperforming both the “treat all” and “treat none” strategies across threshold probabilities of 45–75%.

Following adjustment for age, sex, and tumor stage, the risk score demonstrated independent prognostic value (HR = 1.2, 95% CI 1.08–1.40, *p* = 0.001; Figure 4A). The variable with the largest contribution to the model was tumor stage (Advanced vs. Early; HR = 2.1, *p* < 0.001), and age and sex did not reach significance. The nomogram integrates the three genes (*STC2*, *CKS1B*, *PSAT1*) along with age, sex, and tumor stage—a total of six variables—to visually predict individual mortality risk (Figure 4B). The segment corresponding to tumor stage is the longest, further confirming its role as the primary determinant of prognosis; higher expression levels of *STC2*, *CKS1B*, and *PSAT1* are associated with higher risk scores. The calibration curve shows good agreement between predicted probabilities and actual incidence rates (Figure 4C). Decision curve analysis indicated that the 1-year DCA provided a positive net benefit within a threshold probability range of approximately 10–40%; the effective threshold range for the 3-year DCA broadened to 25–55%; the 5-year DCA performed best, with a net benefit significantly higher than that of the “treat all” and “treat none” strategies within the 45–75% threshold range (Figure 5).

### 3.5. External Validation Confirms the Signature’s Cross-Platform Generalizability

**Figure 6:**
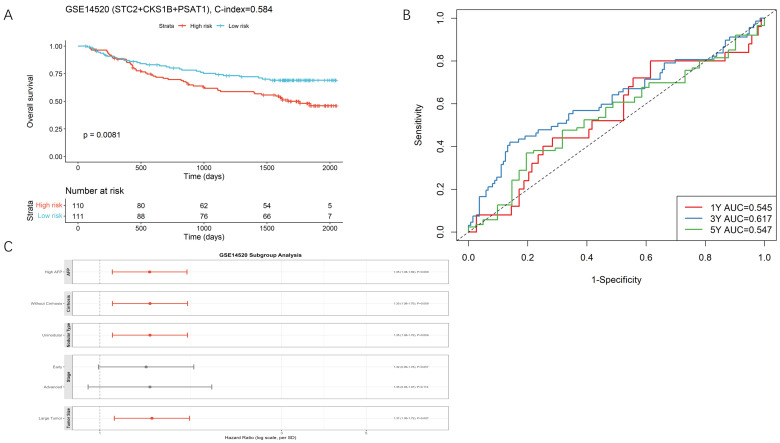
External validation in the GSE14520 cohort. (**A**) Kaplan–Meier estimates of survival for risk-stratified groups. (**B**) Time-dependent receiver operating characteristic curves. (**C**) Forest plot of subgroup-specific hazard ratios, illustrating the prognostic effect of a one standard deviation increase in risk score.

External validation in the independent GSE14520 microarray cohort (*n* = 221) confirmed that the three-gene signature effectively discriminated against patient survival (log-rank *p* = 0.0081, C-index = 0.584; Figure 6A). The 1-year, 3-year, and 5-year AUCs were 0.545, 0.617, and 0.547, respectively (Figure 6B). Subgroup analyses further evaluated the prognostic value of this signature in different clinical contexts (Figure 6C). The results showed that among patients with high AFP (HR = 1.35, *p* = 0.009), no cirrhosis (HR = 1.35, *p* = 0.009), single-nodule type (HR = 1.35, *p* = 0.009), and large tumor (HR = 1.37, *p* = 0.007), a one-standard-deviation increase in the risk score was associated with a 35–37% increase in the risk of death, demonstrating significant predictive power. Among patients with early-stage (HR = 1.32, *p* = 0.057) and advanced-stage (HR = 1.35, *p* = 0.113) disease, the predictive trends of the risk score were consistent but did not reach statistical significance. Due to incomplete staging data, multivariate analysis was not performed in this cohort.

### 3.6. Significant Enrichment of Proliferation-Related Pathways in the High-Risk Group

**Figure 7:**
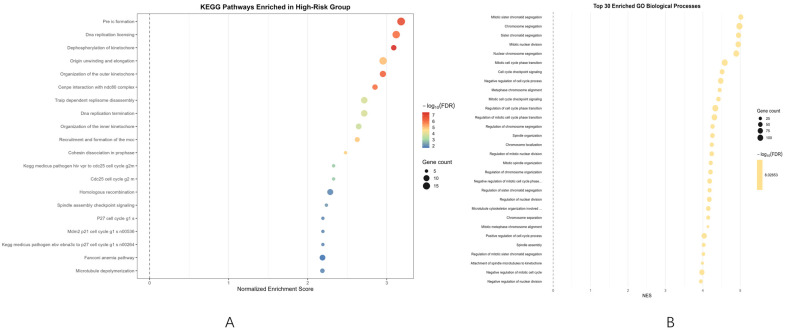
Enriched molecular pathways associated with the high-risk group. (**A**) Cell-cycle pathways—DNA replication licensing, spindle assembly checkpoint, and outer kinetochore organization—were significantly enriched in the high-risk group according to KEGG analysis (NES > 2.1, FDR < 0.05). (**B**) GO biological process enrichment analysis showed the most prominent enrichment in terms such as mitotic sister chromatid separation and chromosome segregation (NES 3.8–5.0).

Gene set enrichment analysis demonstrated that the high-risk group exhibited marked overrepresentation of cell-cycle-associated KEGG pathways, such as DNA replication licensing, spindle assembly checkpoint signaling, and outer kinetochore organization (NES > 2.1, FDR < 0.05; Figure 7A). Key genes driving these enriched pathways included DNA replication initiation factors (*CDC45*, *MCM2–7*) and mitotic checkpoint kinases (*AURKB*, *PLK1*, *BUB1B*). GO analysis further corroborated these findings: terms such as mitotic sister chromatid separation and chromosome segregation showed the most prominent enrichment (NES as high as 3.8–5.0). Core hub genes—*CDK1*, *PLK1*, *AURKA*, and the MCM family—act in a tightly coordinated manner to collectively drive malignant proliferation and chromosomal instability in high-risk cells (Figure 7B). These results are consistent with the poor prognostic clinical characteristics of the high-risk group. See Supplementary File S1 for detailed data tables.

### 3.7. Immune Microenvironment Characteristics Associated with the Three-Gene Signature

**Figure 8:**
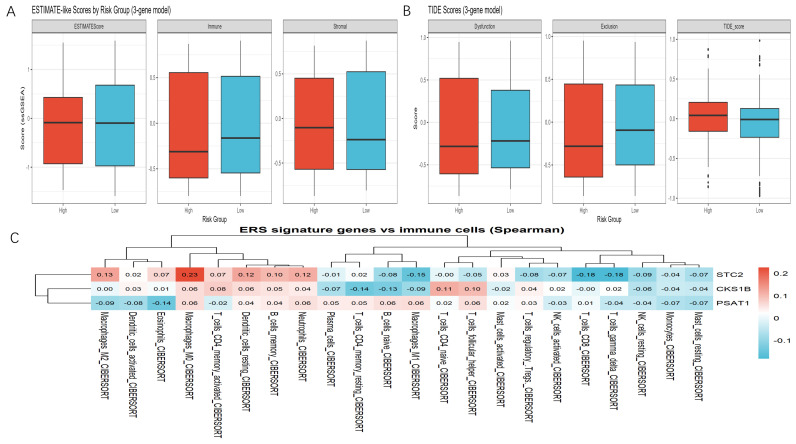
Immune microenvironment characteristics associated with the 3-gene signature. (**A**) ESTIMATE-derived stromal, immune, and composite scores did not differ significantly between risk strata. (**B**) TIDE analysis revealed markedly higher scores in the high-risk group (*p* = 0.019). (**C**) Correlation heatmap of the three signature genes (*STC2*, *CKS1B*, *PSAT1*) with infiltrating immune cell populations.

The Spearman correlation heatmap showed that *STC2* was significantly positively correlated with M0 macrophages (r = 0.23) and significantly negatively correlated with CD8^+^ T cells and γδ T cells (r = −0.18); *CKS1B* and *PSAT1* generally exhibited weak correlations with all immune cell types (Figure 8C). ESTIMATE score analysis indicated no significant differences between the high- and low-risk groups in terms of stromal score, immune score, or composite score (*p* = 0.933, 0.405, 0.839; Figure 8A). TIDE evaluation demonstrated a significantly higher TIDE score in the high-risk group (*p* = 0.019), indicating a greater likelihood of immune escape and a possibly reduced benefit from immune checkpoint blockade. Notably, T-cell exclusion exhibited a marginal trend (*p* = 0.052), whereas T-cell dysfunction did not differ significantly between groups (*p* = 0.638; Figure 8B).

### 3.8. Single-Cell Transcriptomics Reveals Cell-Specific Expression of ERS Genes

**Figure 9:**
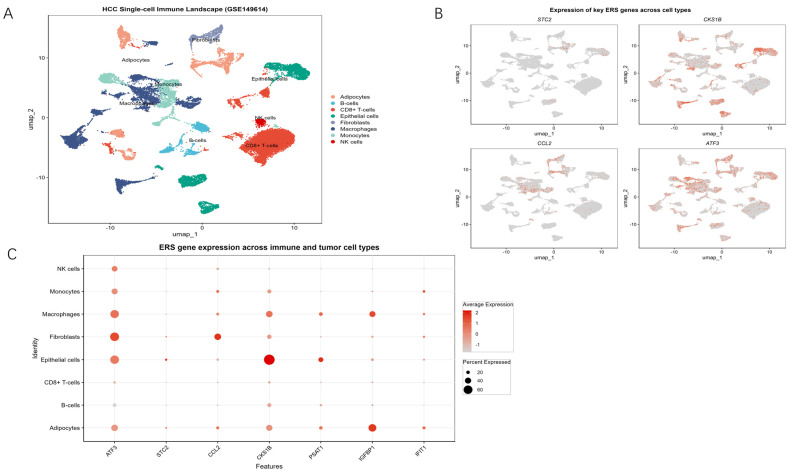
Single-cell transcriptomics reveals cell-specific expression of ERS genes. (**A**) Two-dimensional UMAP embedding of the eight major cell types. (**B**) Expression features of *STC2*, *CKS1B*, *CCL2*, and *ATF3* projected onto the UMAP, highlighting their differential distribution across cell lineages. (**C**) Dot plot summary of ERS-related gene expression per cell type, where color denotes scaled average expression and dot size indicates the fraction of expressing cells.

**Figure 10:**
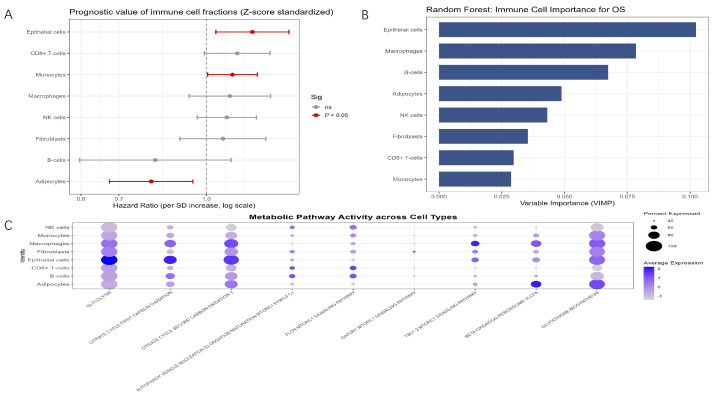
Association of immune cell composition and metabolic activity with prognosis. (**A**) Cox regression forest plot correlating immune cell infiltration proportions with overall survival. Risk factors (HR > 1) and protective factors (HR < 1) are identified; statistically significant results (*p* < 0.05) are shown in red. (**B**) Variable importance measures derived from random survival forest analysis. Higher VIMP indicates stronger influence on survival prediction. (**C**) Distribution of metabolic pathway activities across the eight cell types, highlighting glycolysis, the tricarboxylic acid cycle, and β-oxidation.

Analysis of the GSE149614 single-cell dataset (34,782 cells) identified eight cell types (Figure 9A). Random forest analysis indicated that epithelial cells were the most important cell type for prognostic prediction, and their proportion was significantly associated with poor prognosis (HR > 1, *p* < 0.05; Figure 10A,B). *STC2* and *CKS1B* were expressed almost exclusively in epithelial cells, whereas CCL2 and ATF3 were primarily expressed in macrophages and fibroblasts (Figure 9B,C). The proportion of adipocytes was significantly negatively correlated with the risk of death (HR < 1), and these cells highly expressed IGFBP1 (Figure 9C and Figure 10A). Metabolic pathway analysis revealed that epithelial cells exhibited significant metabolic reprogramming, with significantly higher activity in glycolysis, the tricarboxylic acid cycle, and glutathione synthesis compared to other immune cells. In contrast, adipocytes displayed a unique metabolic profile characterized primarily by fatty acid β-oxidation activity (Figure 10C).

### 3.9. Comparison of Machine Learning Algorithms and Validation of Feature Importance

**Figure 11:**
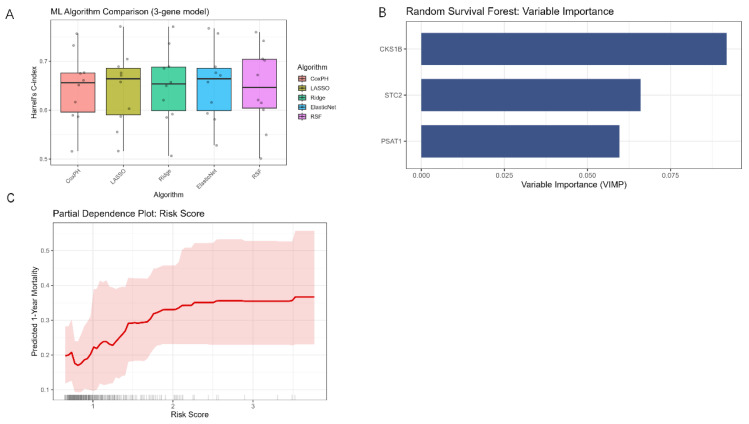
Comparison of machine learning algorithms and validation of feature importance. (**A**) Box plots of the C-index for 10-fold cross-validation of the five algorithms. (**B**) Ranking of variable importance based on a random survival forest using three genes. (**C**) Partial dependence plot of the risk score; the pink shading represents the 95% confidence interval, and the rug plot at the bottom shows the distribution of samples in the training set.

A 10-fold cross-validation of five machine learning algorithms showed that the median Harrell’s C-index for each algorithm remained stable at around 0.65 (Figure 11A). A variable importance analysis based on a random survival forest model using three genes revealed that *CKS1B* contributed most to prognostic prediction, followed by *STC2*, while *PSAT1* had the weakest contribution (Figure 11B). A partial dependence plot illustrates the relationship between the risk score and the predicted 1-year mortality rate (Figure 11C). As the risk score increases, the predicted mortality rate rises; however, the curve plateaus when the score exceeds 2.5, and the confidence interval widens. The predicted 1-year mortality rate for the lowest-risk group (score approximately 0.8) was approximately 18–20%. The bottom rug plot shows that samples are primarily concentrated in the 0.5–2.0 range, with sparse data for scores > 2.5.

### 3.10. Drug Sensitivity Analysis to Identify Potential Therapeutic Agents

**Figure 12:**
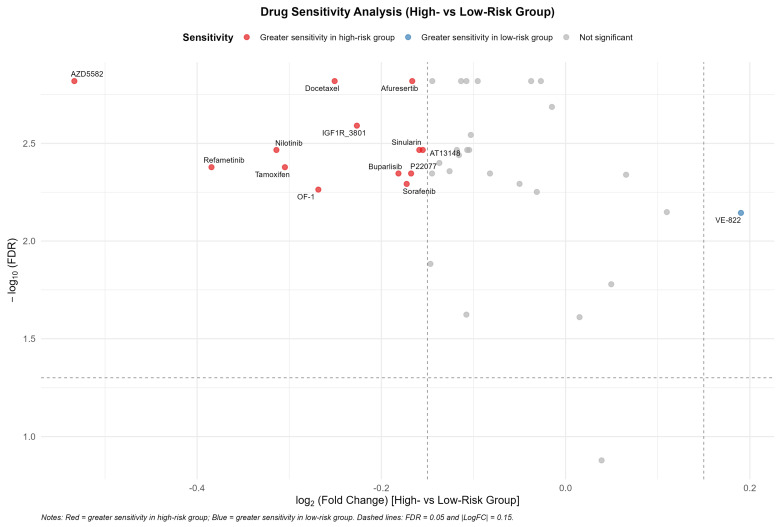
Drug sensitivity volcano plot. The x-axis denotes the log_2_-transformed IC_50_ ratio (high-risk/low-risk). Negative values (red) indicate greater sensitivity in the high-risk group, while positive values (blue) indicate greater sensitivity in the low-risk group. The dashed lines correspond to the significance thresholds FDR = 0.05 and |LogFC| = 0.15.

IC_50_ values for 286 drugs in the TCGA cohort were predicted using Ridge regression. Analysis of differences between the high- and low-risk groups revealed that the high-risk group exhibited greater sensitivity to multiple drugs (Figure 12). Among these, AZD5582 exhibited the largest effect size (LogFC = −0.53, FDR < 0.001), followed by refametinib (LogFC = −0.38, FDR = 0.004), nilotinib (LogFC = −0.31, FDR = 0.003), tamoxifen (LogFC = −0.30, FDR = 0.004), and docetaxel (LogFC = −0.25, FDR = 0.002). In the low-risk group, only VE-822 met the criteria of |LogFC| > 0.15 and FDR < 0.05 (LogFC = 0.19, FDR = 0.007). Overall, the effect sizes were generally small.

## 4. Discussion

In this study, we constructed a three-gene prognostic signature consisting of *STC2*, *CKS1B*, and *PSAT1* by screening an endoplasmic reticulum stress-associated ceRNA network. The following sections discuss its biological plausibility, cross-platform performance, immune microenvironment context, and therapeutic implications.

### 4.1. Comparison with Previous Studies

The functions of *STC2*, *CKS1B*, and *PSAT1* in tumors have been previously reported. *STC2*, as an endoplasmic reticulum stress-induced glycoprotein, drives HCC progression by inhibiting apoptosis and promoting epithelial–mesenchymal transition [46]. As comprehensively reviewed by Qie and Sang, *STC2* is significantly up-regulated under ER stress conditions and prevents apoptosis; mechanistically, *STC2* promotes EMT by up-regulating mesenchymal markers *N-cadherin* and *vimentin* while suppressing the epithelial marker *E-cadherin*, and also enhances cell invasion by up-regulating matrix metalloproteinase MMP-2 and MMP-9. Furthermore, elevated *STC2* expression is correlated with tumor invasion, metastasis, and poor prognosis in HCC patients, and *STC2* has been implicated in mediating resistance to multiple chemotherapeutic agents [46]. A recent study by Zhou et al. further demonstrated that *STC2* is significantly up-regulated in sorafenib-resistant HCC cells and that siRNA-mediated inhibition of *STC2* potentiates sorafenib sensitivity in HCC cell lines, providing direct evidence for the role of *STC2* in sorafenib resistance in HCC [47]. *CKS1B* promotes proliferation and invasion by activating the JAK/STAT3 pathway [48]; functional studies have confirmed that *CKS1B* knockdown significantly inhibits the migration and invasion of HCC cells, and its overexpression is associated with clinical invasiveness and metastatic potential in HCC [48]. *PSAT1*, regulated by lncRNA, is involved in serine metabolism and metastasis [49]; specifically, *PSAT1* has been demonstrated to promote HCC cell proliferation and metastasis, and rescue experiments have confirmed that *PSAT1* is essential for HCC cell growth, migration, and invasion [49]. Unlike the aforementioned single-gene studies, this study is the first to integrate these three genes into a prognostic signature from the perspective of ERS-related ceRNA networks and to validate it across platforms in GSE14520, demonstrating the complementary value of multigene combinations.

In terms of model building paradigms, this study is similar to the four-gene signature based on epigenetically dysregulated genes reported by Zhang et al. [50], but it has the following characteristics: ① it contains only three genes, facilitating clinical translation; ② it is rooted in ceRNA regulatory axes such as *KCNQ1OT1*/miR-485-5p/*STC2*, providing an upstream mechanistic context; ③ single-cell analysis confirmed the tumor epithelial cell origin of the core genes; ④ it established a complete chain of evidence spanning ceRNA networks, single-cell localization, external validation, and drug sensitivity prediction. These characteristics give this signature greater translational potential compared with single-level prognostic models. However, the synergistic regulatory mechanism of these three genes in endoplasmic reticulum stress still requires further validation through in vitro experiments.

### 4.2. Challenges of Cross-Platform Validation

The three-gene signature developed in this study maintained significant prognostic The three-gene signature developed in this study maintained significant prognostic stratification ability in the independent microarray cohort GSE14520, but its C-index decreased from 0.653 in the training set to 0.584. Issues regarding the cross-platform compatibility of transcriptomic data have been widely documented [51]; one study further demonstrated that even when using optimal cross-platform normalization methods, model performance declines when the training and validation sets originate from different technical platforms [52]. This provides a mathematical explanation for the inevitable decline in the C-index of prognostic models trained on RNA-seq data when applied to external microarray cohorts. Furthermore, although the lncRNA *AC145207.5*, identified during the initial ceRNA network screening, demonstrated some prognostic value in the TCGA training set, it could not be detected in GSE14520 due to the inherent limitations of microarray platforms in lncRNA probe coverage and was therefore excluded from the final model. This technical limitation also reflects the practical constraints on biomarker selection during cross-platform validation. Differences between the two cohorts in patient ethnicity, etiological composition, and clinical staging distribution may also have contributed to the partial decline in performance. Despite this decline, the three-gene signature maintained significant prognostic stratification ability in GSE14520, with a log-rank *p* value of 0.0081, suggesting its value as a tool for relative risk stratification rather than absolute survival prediction. Clinically, this signature could be applied to identify high-risk patients who may benefit from more intensive postoperative surveillance, such as imaging every 3 months, or from prioritized enrollment in adjuvant therapy clinical trials. The median risk score derived from the TCGA training cohort is proposed as the threshold for dichotomizing patients into high- and low-risk groups. This signature should be considered an exploratory tool at this stage, and its clinical translation requires validation in a multicenter, prospective cohort using a standardized testing platform.

### 4.3. Single-Cell Analysis and the Immune Microenvironment

Through single-cell transcriptomic analysis, this study provided a microenvironmental context for the three-gene signature at the cellular level. *STC2* and *CKS1B* are expressed almost exclusively in tumor epithelial cells, while *CCL2* and *ATF3* primarily originate from macrophages and fibroblasts, indicating that the signature captures both intrinsic tumor characteristics and microenvironmental stromal signals. Random forest analysis revealed that epithelial cells and macrophages are the two cell types that contribute most significantly to prognostic prediction. This is consistent with reports that TREM-1^+^ tumor-associated macrophages accumulate in the late stages of HCC progression and promote immune suppression by inducing CD8^+^ T cell dysfunction [53]. Adipocytes exhibited an independent protective trend and highly expressed *IGFBP1*; however, given their low abundance and small variance in MuSiC deconvolution, this finding should be regarded as a hypothesis-generating observation that requires validation in a larger cohort with higher adipocyte content or through targeted single-cell studies. Single-cell metabolic pathway analysis revealed that tumor cells rely primarily on glycolysis, whereas adipocytes favor β-oxidation, indicating a metabolic division of labor among different cell populations. These findings collectively suggest that the three-gene signature not only reflects the proliferative activity of tumor cells but is also associated with the compositional characteristics of the immune microenvironment.

Although our drug sensitivity analysis did not include immunotherapeutic agents, our immune microenvironment findings provide insights into the potential value of immunotherapy. TIDE analysis revealed a significantly higher TIDE score in the high-risk group, suggesting that these patients may derive less benefit from immune checkpoint inhibitor monotherapy such as anti-PD-1/PD-L1, which is consistent with the observed decline in CD8^+^ T cell infiltration. This finding aligns with evidence that multiple resistance mechanisms, including an immunosuppressive tumor microenvironment characterized by enrichment of regulatory T cells and myeloid-derived suppressor cells, can limit the efficacy of immune checkpoint inhibitors in HCC [54]. Therefore, combination strategies that target both the immunosuppressive microenvironment and tumor cell-intrinsic vulnerabilities may be more effective for high-risk patients. Furthermore, the enhanced sensitivity of the high-risk group to cell-cycle/mitosis-targeting agents such as docetaxel raises the possibility that chemotherapy combined with immunotherapy may trigger immunogenic cell death and enhance antitumour immunity [54]. However, TIDE analysis provides only an in silico inference, and these hypotheses require validation in prospective cohorts with immunotherapy response data before any clinical application can be considered.

### 4.4. Implications for Drug Sensitivity

Through drug sensitivity analysis, this study provides preliminary drug leads for the clinical translation of the three-gene signature. The high-risk group exhibited greater sensitivity to multiple drugs, with AZD5582 showing the greatest effect, followed by docetaxel, nilotinib, and refametinib, among others. In particular, the sensitivity to the mitotic inhibitor docetaxel is directly consistent with the GSEA findings showing significant enrichment of pathways such as the spindle assembly checkpoint and DNA replication in the high-risk group. Additionally, sensitivity to agents such as AZD5582, nilotinib, and refametinib suggests that high-risk tumors may concurrently depend on multiple pro-survival and proliferative signaling cascades, forming a coherent logical loop from molecular mechanisms to potential therapeutic strategies. In the low-risk group, the ATR inhibitor VE-822 demonstrated a significant but smaller increase in sensitivity, suggesting that patients in different risk strata may benefit from distinct targeted therapeutic strategies. It is important to distinguish between statistical significance and biological effect size in these findings. The extremely low FDR values indicate that the observed differences in drug sensitivity between risk groups are robust and non-random; however, the small absolute LogFC values suggest that these differences may reflect only modest shifts in drug sensitivity rather than strong selective effects against specific agents. Therefore, these results should be regarded as hypothesis-generating leads for future investigation rather than immediate therapeutic recommendations. Furthermore, there is a discrepancy between IC_50_ predictions based on GDSC cell lines and actual in vivo drug efficacy—as the GDSC team has pointed out, in vitro cancer cell line models have inherent limitations [52,55]. The clinical applicability of these findings requires validation in HCC cell lines and animal models before any therapeutic recommendations can be considered.

### 4.5. Limitations

This study has several limitations. First, although the three-gene signature maintained significant prognostic stratification ability in GSE14520, its C-index decreased from 0.653 in the training set to 0.584, representing a predictable decline in absolute predictive accuracy commonly observed in cross-platform transcriptomic biomarker studies. The robust stratification was nevertheless confirmed by a significant log-rank *p* = 0.008. Furthermore, the three-gene signature was validated in only one external cohort, GSE14520. Although the consistent prognostic stratification observed in both the TCGA training set and GSE14520 provides preliminary evidence of cross-platform generalizability, additional validation in larger, independent HCC cohorts with complete gene coverage is warranted to further strengthen the robustness of these findings. Second, the predicted 1-year mortality rate for the lowest risk group remained close to 18–20%, indicating that the model exhibits bias in absolute risk estimation; it is therefore more suitable for relative risk stratification among patients rather than for the precise prediction of individual absolute mortality probabilities. Third, although the protective trend observed in adipocytes in single-cell analysis is insightful, their abundance is low and variance is small in MuSiC deconvolution; the robustness of this finding requires further evaluation in a larger cohort. Fourth, the drug sensitivity analysis was based on IC_50_ predictions from GDSC cell lines, with generally small effect sizes; in vitro cancer cell line models have inherent limitations, and their clinical applicability requires validation through in vitro experiments and in vivo models. Fifth, the synergistic regulatory mechanism of *STC2*, *CKS1B*, and *PSAT1* in endoplasmic reticulum stress has not yet been experimentally validated; how these three genes interact to drive HCC proliferation, metabolic reprogramming, and immune evasion remains to be elucidated by in-depth studies of molecular mechanisms. Finally, the current drug sensitivity analysis was limited to compounds in the GDSC database and did not evaluate the predictive value of the three-gene signature for immunotherapy response; future studies should incorporate cohorts treated with immune checkpoint inhibitors to address this gap. In addition, the lncRNA *AC145207.5* and the gene *H2AX*, identified during the initial ceRNA network screening, were excluded from the final model because their probes were absent from the GSE14520 microarray platform. Due to current limitations in sample availability and resources, qPCR validation of the complete ceRNA regulatory axis containing these molecules could not be performed in this study. Future independent validation of the complete ceRNA regulatory axis containing *AC145207.5* using qPCR or RNA-seq platforms will help further confirm the reliability of constructing prognostic signatures based on ceRNA networks.

### 4.6. Conclusions and Outlook

By integrating transcriptomic, single-cell transcriptomic, and drug sensitivity data, and based on a screening of endoplasmic reticulum stress-related ceRNA networks, this study constructed a three-gene prognostic signature comprising *STC2*, *CKS1B*, and *PSAT1*. This signature demonstrated independent prognostic stratification capabilities in both the TCGA training set and the independent microarray cohort GSE14520. It also revealed molecular characteristics centered on cell-cycle dysregulation and an immunosuppressive microenvironment in the high-risk group, providing an exploratory tool for prognostic assessment of HCC.

Future research could be advanced in the following areas. First, the generalizability of this signature needs to be validated in additional independent cohorts and in a multicenter, prospective cohort using a standardized testing platform to overcome the current bottleneck of reduced performance across different platforms. Second, as the core driver gene of this signature, *CKS1B* warrants in-depth molecular biological research into its specific regulatory mechanisms in HCC and its functional association with endoplasmic reticulum stress. Furthermore, integrating multimodal data—including clinical staging, AFP, and radiomics—to construct a composite model incorporating clinical variables holds promise for surpassing the current performance ceiling of approximately 0.65 for the C-index. Preliminary results from drug sensitivity analysis provide potential therapeutic clues for patients in the high-risk group and warrant further experimental validation. Single-cell analysis suggests that adipocytes may play a protective role in the HCC microenvironment, but the specific mechanism still needs to be elucidated through functional experiments. Lastly, the lncRNA *AC145207.5* identified in the initial ceRNA network could not be evaluated in the current external validation cohort due to the absence of probes on the microarray platform. Future independent validation of the complete ceRNA regulatory axis containing this lncRNA using qPCR or RNA-seq platforms will help further confirm the reliability of the strategy of constructing prognostic signatures based on ceRNA networks. Pending further validation, the signature may serve as an adjunctive tool for identifying patients who warrant closer postoperative monitoring.

## Data Availability

The TCGA-LIHC transcriptomic data and clinical information used in this study were obtained from The Cancer Genome Atlas (https://portal.gdc.cancer.gov). The external validation dataset GSE14520 was obtained from the Gene Expression Omnibus (GEO, https://www.ncbi.nlm.nih.gov/geo). The single-cell transcriptomic dataset GSE149614 was obtained from the GEO database. The drug sensitivity data were obtained from the Genomics of Drug Sensitivity in Cancer (GDSC, https://www.cancerrxgene.org). The endoplasmic reticulum stress-related gene sets were obtained from the Molecular Signatures Database (MSigDB, https://www.gsea-msigdb.org). All analysis codes and resulting data generated in this study are available from the corresponding author upon reasonable request.

## Supplementary Materials

The following supporting information can be downloaded at: https://www.mdpi.com/article/10.3390/cimb48070743/s1.

## Abbreviations

TCGA: The Cancer Genome Atlas, GEO: Gene Expression Omnibus, GDSC: Genomics of Drug Sensitivity in Cancer, MSigDB: Molecular Signatures Database, HCC: Hepatocellular Carcinoma, ERS: Endoplasmic Reticulum Stress, ceRNA: Competing Endogenous RNA, lncRNA: Long Non-coding RNA, miRNA: MicroRNA, mRNA: Messenger RNA, DEG: Differentially Expressed Gene, GSEA: Gene Set Enrichment Analysis, KEGG: Kyoto Encyclopedia of Genes and Genomes, GO: Gene Ontology, NES: Normalized Enrichment Score, FDR: False Discovery Rate, OS: Overall Survival, C-index: Concordance Index, HR: Hazard Ratio, CI: Confidence Interval, AUC: Area Under the Curve, ROC: Receiver Operating Characteristic, DCA: Decision Curve Analysis, KM: Kaplan–Meier, LASSO: Least Absolute Shrinkage and Selection Operator, VIMP: Variable Importance, PDP: Partial Dependence Plot, PCA: Principal Component Analysis, UMAP: Uniform Manifold Approximation and Projection, MuSiC: Multi-subject Single Cell, TIDE: Tumor Immune Dysfunction and Exclusion, TME: Tumor Microenvironment, LogFC: Log Fold Change, AFP: Alpha-fetoprotein, ssGSEA: Single-sample Gene Set Enrichment Analysis.
